# Next-Generation Sequencing-Based Copy Number Variation Analysis in Chinese Patients with Primary Ciliary Dyskinesia Revealed Novel *DNAH5* Copy Number Variations

**DOI:** 10.1007/s43657-023-00130-0

**Published:** 2024-02-22

**Authors:** Weicheng Chen, Zhuoyao Guo, Mengru Li, Wei Sheng, Guoying Huang

**Affiliations:** 1 Cardiovascular Center, Children’s Hospital of Fudan University, 399 Wanyuan Road, Shanghai, 201102 China; 2 Respirology Department, Children’s Hospital of Fudan University, Shanghai, 201102 China

**Keywords:** Primary ciliary dyskinesia, *DNAH5* gene, Copy number variation, Phenotypic heterogeneity

## Abstract

Primary ciliary dyskinesia (PCD) is a rare disorder characterized by extensive genetic heterogeneity. However, in the genetic pathogenesis of PCD, copy number variation (CNV) has not received sufficient attention and has rarely been reported, especially in China. Next-generation sequencing (NGS) followed by targeted CNV analysis was used in patients highly suspected to have PCD with negative results in routine whole-exome sequencing (WES) analysis. Quantitative real-time polymerase chain reaction (qPCR) and Sanger sequencing were used to confirm these CNVs. To further characterize the ciliary phenotypes, high-speed video microscopy analysis (HSVA), transmission electron microscopy (TEM), and immunofluorescence (IF) analysis were used. Patient 1 (F1: II-1), a 0.6-year-old girl, came from a nonconsanguineous family-I. She presented with situs inversus totalis, neonatal respiratory distress, and sinusitis. The nasal nitric oxide level was markedly reduced. The respiratory cilia beat with reduced amplitude. TEM revealed shortened outer dynein arms (ODA) of cilia. chr5:13717907-13722661del spanning exons 71–72 was identified by NGS-based CNV analysis. Patient 2 (F2: IV-4), a 37-year-old man, and his eldest brother Patient 3 (F2: IV-2) came from a consanguineous family-II. Both had sinusitis, bronchiectasis and situs inversus totalis. The respiratory cilia of Patient 2 and Patient 3 were found to be uniformly immotile, with ODA defects. Two novel homozygous deletions chr5:13720087_13733030delinsGTTTTC and chr5:13649539_1 3707643del, spanning exons 69–71 and exons 77–79 were identified by NGS-based CNV analysis. Abnormalities in DNA copy number were confirmed by qPCR amplification. IF showed that the respiratory cilia of Patient 1 and Patient 2 were deficient in dynein axonemal heavy chain 5 (DNAH5) protein expression. This report identified three novel *DNAH5* disease-associated variants by WES-based CNV analysis. Our study expands the genetic spectrum of PCD with *DNAH5* in the Chinese population.

**Supplementary Information:**

The online version contains supplementary material available at 10.1007/s43657-023-00130-0.

## Introduction

Primary ciliary dyskinesia (PCD) is a rare, heterogeneous ciliopathy resulting in mucociliary clearance failure. Patients typically present with newborn respiratory distress, daily wet cough, chronic nasal congestion, and laterality defects. PCD diagnosis can be achieved by following diagnostic algorithms that include nasal nitric oxide (nNO) measurements, high-speed video microscopy analysis (HSVA), immunofluorescence (IF), transmission electron microscopy (TEM), and molecular testing (Lucas et al. 2017). Currently, approximately, 50 genes are known to be associated with PCD (Wallmeier et al. 2020). Due to a lack of appropriate diagnostic facilities and trained diagnosticians, few PCD patients have been reported in China (Guan et al. 2021).

Dynein axonemal heavy chain 5 (DNAH5) is a protein that functions as a force-generating protein to produce cilia bending (Olbrich et al. 2002). It has been reported that *DNAH5* is responsible for nearly 15–29% of all cases of PCD in European and American populations (Zariwala et al. 1993). Previous studies focused on patients with *DNAH5* in non-Asian populations, revealing that mutations in *DNAH5* lead to absent or shorter outer dynein arms (ODA) in respiratory cilia that are mostly immotile or only exhibit flickering movements (Raidt et al. 2014; Hornef et al. 2006; Emiralioğlu et al. 2020).

At least 100 different pathogenic variants in the *DNAH5* gene have been reported (Zariwala et al. 1993; Hornef et al. 2006; Emiralioğlu et al. 2020; Wang et al. 2021). However, the clinical significance of copy number variations (CNVs) in *DNAH5* has rarely been reported. Moreover, CNV analysis in PCD in the Chinese population has never been examined. CNVs contribute substantially to autosomal recessive Mendelian disorders. CNVs have been reported in up to 10.8% of autosomal recessive Mendelian disorders but are not detectable by routine whole-exome sequencing (WES) analysis (Aradhya et al. 2012). In this study, we performed WES analysis to identify disease-causing variants. For “negative” cases, WES-based CNV analysis was conducted to detect CNVs. Eventually, three PCD patients in the Chinese population carrying three novel *DNAH5* CNVs were identified. Sanger sequencing, quantitative real-time polymerase chain reaction (qPCR) and IF were performed to further support pathogenicity.

## Materials and Methods

### Patients and Clinical Materials

The project was approved by the Ethics Committee of The Children's Hospital of Fudan University, and written informed consent was acquired from the guardians of the patients participating in this study. Three affected individuals and five relatives from two families were recruited for this study. Clinical data, including lung function, high-resolution computed tomography (HRCT), bacterial cultures, and PCD-related diagnostic tests, were obtained at enrollment. At the same time, clinical evaluations and genetic testing were performed in five relatives, including the patients' parents and Patient 3's daughter (Table S1). The diagnosis of PCD was based on clinical findings, nNO, HSVA, TEM, and genetic testing, in accordance with the guidelines of the European Respiratory Society (Lucas et al. 2017).

### nNO Measurements

Measurements of nNO were performed with an EcoMedics CLD88 chemiluminescence NO analyser (Duernten, Switzerland); the aspiration sampling rate of 330 nL/min was verified before and after each subject was tested. The measurement of nNO in cooperative children was performed by breath hold maneuver for at least 10 s to close their velum as described previously (Guo et al. 2020). For children who were uncooperative (less than five years old), nasal sampling was performed for 60 s during tidal breathing. The results were reported in nL/min with the following equation: nNO (nL/min) = NO (ppb) × sampling rate (nL/min).

### TEM and HSVA

Nasal tissue was collected using a Rhino-Probe (Arlington Scientific, Springville, UT). To avoid secondary ciliary dyskinesia, nasal samples were taken in patients who were free from acute airway infection for at least four weeks. The tissue was suspended in L-15 medium (Invitrogen, CA) immediately for analysis using a Leica inverted microscope (Leica DMI300B, Solms, Germany) with a 63 × oil objective under differential interference contrast optics. Cilia beats were recorded at 200 frames/s at room temperature (25 °C) using a 680 PROSILICA GE camera (Allied Vision, PA). At least 10 videos were derived from each sample. The digital recordings were evaluated by two experienced but blinded investigators.

The ciliary ultrastructure was analyzed in nasal tissue fixed in 2.5% glutaraldehyde, as described previously (Guo et al. 2020). For each specimen, at least 30 transverse ciliary sections of different cells were used to evaluate the internal axonemal structure. Ciliary abnormalities were defined as the presence of defects in > 50% of cilia.

### WES Analysis

Blood samples were obtained from the proband and available family members. Genomic deoxyribonucleic acid (DNA) was extracted using the Gene Blood DNA Rapid Extraction Kit (Qiagen, China). WES was performed by Gemple Biotech Co., Ltd. (Shanghai, China). Whole-exome libraries were constructed using the KAPA platform and KAPA Hyper Prep kit (Roche KAPA, Switzerland). Exomes were captured using SeqCap EZ MedExome (Roche NimbleGen) and sequenced by a HiSeq X Ten instrument (Illumina, San Diego, CA). Raw data were evaluated using FASTQC (version 0.11.5), and the linker sequences were removed by Cutadapt (version 1.10). BWA software (version 0.7.15) was used to align the reads to the human reference genome GRCh37/hg19 (UCSC). Variant filtering was conducted according to variant type, pathogenicity predictor scores, and variant frequencies in population databases. Briefly, allelic variants with a frequency ≥ 5% in any of the databases used (GnomAD, ExAC and 1000 Genomes) were filtered out. Variants classified as benign or probably benign by multiple subscribers in ClinVar database, synonymous variants and intronic variants localized more than 10 nucleotides from the exon/intron junction were also filtered out, nonsynonymous missense variants with a “benign effect” according from all pathogenicity predictors (SIFT, PolyPhen, MutationTaster, CADD) were excluded. The remaining variants were further assessed according to the inheritance model, available report on the pathogenicity, and recorded clinical manifestation. Variants were classified following the American College of Medical Genetics and Genomics (ACMG) guidelines (Richard et al. 2015). Sanger sequencing was performed to validate the candidate variants, and segregation analyses were performed in family members. The RefSeq accession numbers of the transcript and corresponding protein isoform used for mutation nomenclature were NM_001369.2 and NP_001360.1, respectively.

### CNV Analyses

CNV detection based on targeted capture-based next-generation sequencing (NGS) data was performed using the R package panel copy number estimation by a mixture of Poissons (cn.MOPS) (Povysil et al. 2017) set to default parameters. The panelcn.MOPS package is based on the genome-wide and whole-exome-wide CNV detection tool cn.MOPS (Klambauer et al. 2012). cn.MOPS builds a local model that captures the read characteristics of each region of interest, avoiding bias induced by the targeting procedure (Povysil et al. 2017). Twenty-five blood samples that were sequenced using the same targeted panel and did not show any CNVs in complementary array analysis were used as the control dataset. The complete target regions of all target genes of the test and control samples were used for normalization and as controls for the panelcn.MOPS pipeline. Probes spanning an individual region of more than 300 nucleotides were subdivided into smaller targets of at least 70–100 nucleotides and a maximum of 200 nucleotides to increase the resolution of CNV detection. In addition, a csv file was created for each sample, displaying statistical parameters and copy number changes (CN: CN0 = loss; CN1 = one copy; CN2 = two copies; CNx = x copies) for each target.

An average of 10 Gb raw data (fastq) as input was generated for each sample by illumina sequencers. First, the paired-end reads was performed quality control by FASTQC version 0.11.5. Second, Burrows–Wheeler Aligner (BWA) version 0.7.15 (Li and Durbin 2009) was used to align sequencing reads to the reference genome GRCh37. SAM format files were generated by BWA. Third, the SAM format files were further processed to BAM files using Samtools version 1.3.1 (Li et al. 2009), then removing duplicates by Picard version 2.5 (http://broadinstitute.github.io/picard/). After these processes, variant calling was performed by GATK version 3.5 (McKenna et al. 2010) and the vcf file was generated. Finally, we used an in-house software to annotate the variants from the vcf file and integrate information from multiple databases. The final variants can feed to the downstream advanced analysis pipeline.

### qPCR Analysis

To confirm CNVs, qPCR was performed using DNA from patients, their parents and two healthy controls of similar age with Patient 1 and Patient 2. DNA was extracted from peripheral blood. qPCR was prepared using KAPA SYBR FAST qPCR Kit Master Mix (2×) Universal (Roche KAPA, Switzerland) on a CFX96™ Real-Time System instrument (BIO-RAD) and was performed on a LightCycler 480 System II (Roche Diagnostics) in triplicate. The reaction conditions were set at 95 °C for 3 min, followed by 40 cycles of 95 °C for 5 s and 55 °C for 30 s. Specific primers designed by NCBI (https://www.ncbi.nlm.nih.gov/) are summarized in Table S2. The comparative ΔΔCt method was used to calculate relative expression with data normalized to the mean level of an internal standard.

### Whole-Genome Sequencing (WGS)

Genomic DNA was obtained and sheared into fragments with an average length of 350 bp to construct libraries using the KAPA platform and KAPA Hyper Prep kit (Roche KAPA, Switzerland). The libraries were then subjected to sequencing on the Illumina HiSeq X platform, and 150 bp paired-end reads were generated. The same bioinformatics pipeline described above for the exome sequencing analysis was used to analyze *DNAH5* mutations and precise breakpoints.

### IF Analysis

Nasal tissue, obtained by nasal brushing biopsy, was suspended in cell culture medium, spread onto glass slides, and then air dried. Samples were treated with 4% paraformaldehyde, 0.2% Triton-X 100 and 1% BSA before incubation with primary (overnight at 4 °C) and secondary (2 h at room temperature) antibodies. Rabbit polyclonal anti-DNAH5 (HPA037470, Sigma, Sweden) and mouse monoclonal anti-alpha tubulin (ab24610, Abcam, UK) antibodies were used at a dilution of 1:1000. Highly cross-adsorbed secondary antibodies, goat anti-mouse Alexa Fluor 488 (1:1000) and goat anti-rabbit Alexa Fluor 647 (1:1000), were obtained from Abcam. Confocal images were taken using a Leica TCS SP8 confocal laser scanning microscope (Leica, Jena, Germany).

## Results

### Clinical Data

**Fig. 1:**
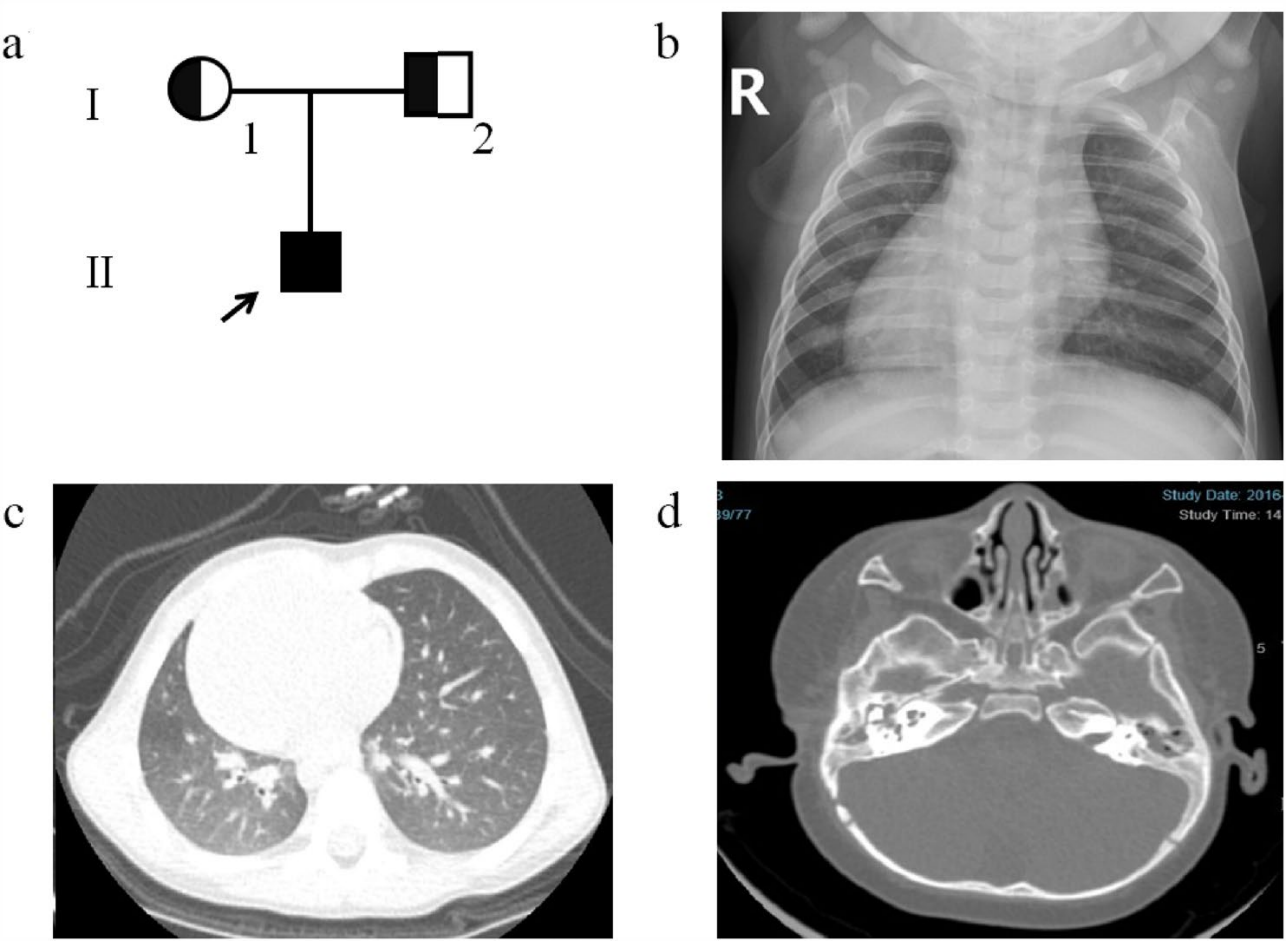
Pedigree structure and clinical examinations of Patient 1's family. **a** Pedigree structure of Patient 1's family. The arrow denotes proband Patient 1. The affected patient is designated by a black symbol; the half-black symbol indicates heterozygous deletions of *DNAH5*. Chest X-ray (CXR) images show mirrored distributed viscera (**b**). CT images of Patient 1 show no abnormality except for dextrocardia (**c**). CT of the paranasal sinus shows otitis media and maxillary sinusitis (**d**)

A 0.6-year-old female (II-1) from Family 1 (Fig. 1a) was referred to our clinic with a chief complaint of persistent wet cough and nasal congestion. Situs inversus totalis was detected during the fetal period (Fig. 1b). The girl was born naturally at full term but displayed shortness of breath starting after a few hours of life and needed supplemental oxygen. No obvious abnormality was noticed on CT scans of the lung (Fig. 1c). A CT scan of the paranasal sinus showed maxillary sinusitis and otitis media (Fig. 1d). Nasal NO production was 8.1 nl/min, which is low and compatible with PCD.

**Fig. 2:**
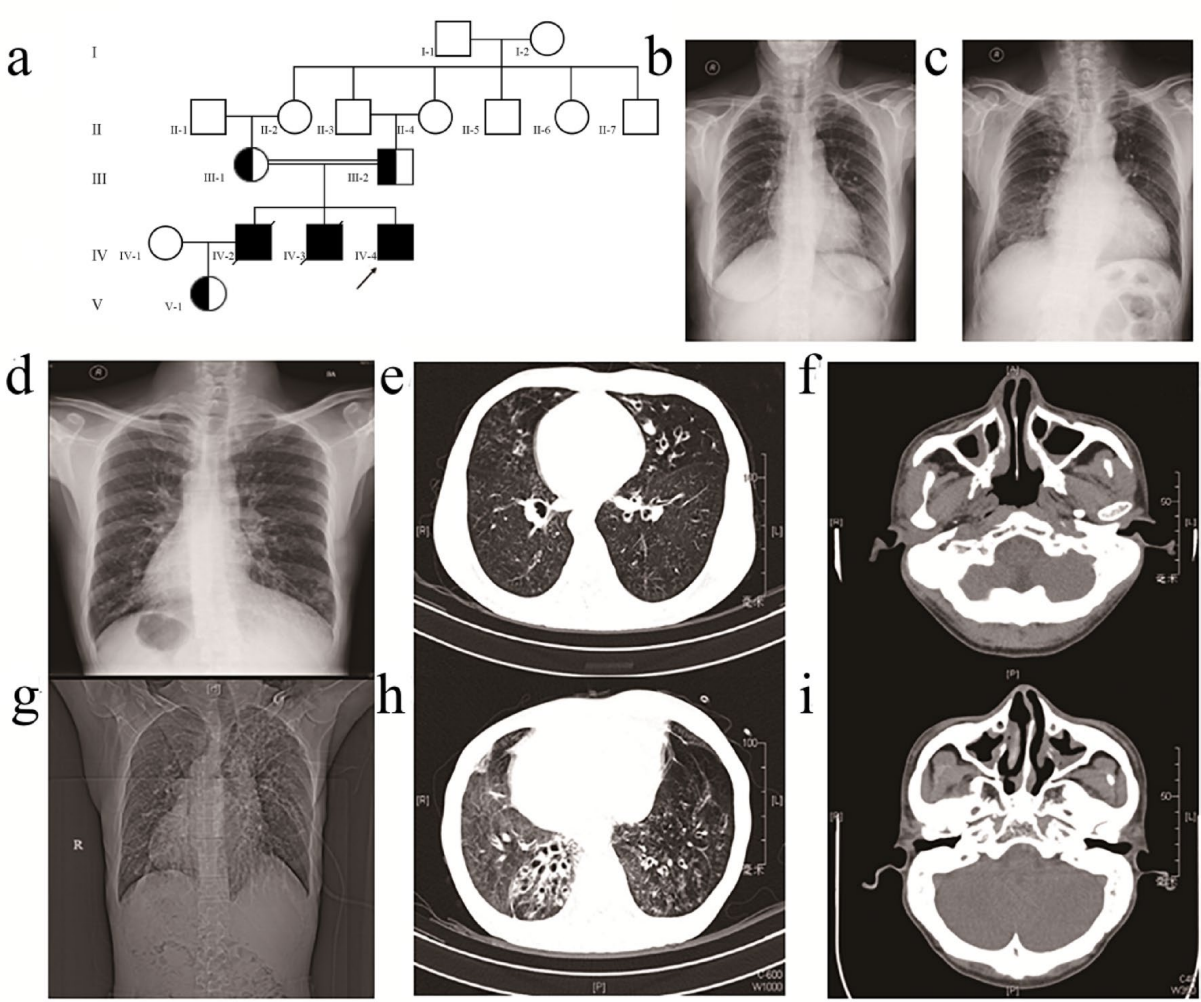
Pedigree structure and clinical examinations of Patient 2's family. **a** Pedigree structure of Patient 2's family. The arrow denotes proband Patient 2. Symbols with diagonal slashes denote deceased individuals. CXR images of the mother (**b**) and father (**c**) of the proband show normal distribution of organs. Clinical imaging of Patient 2 shows mirrored distributed viscera (**d**) and features of bronchiectasis (**e**) and paranasal sinusitis (**f**). Clinical imaging of Patient 2's brother (F2: IV-3) shows distinctive situs inversus (**g**) and more severe bronchiectasis (**h**) and paranasal sinusitis (**i**)

In Family 2, Patient 2 (IV-4) was a 39-year-old male, an offspring of a consanguineous family (Fig. 2a). The proband did not suffer from neonatal respiratory distress during the newborn period or recurrent respiratory diseases during childhood. Otitis and hearing loss were not reported. However, he suffered from productive cough and recurrent pneumonia since adolescence. In addition, the proband was diagnosed with infertility. Chest X-ray (CXR) showed that the cardiac apex was rightward (Fig. 2d). HRCT showed a tree-in-bud pattern and mild bronchiectasis without significant patchy shadow (Fig. 2e). nNO testing of Patient 2 revealed an nNO production rate of 9.9 nL/min. Two siblings of Patient 2 were also affected. The eldest brother, Patient 3 (IV-2), was subjected to recurrent respiratory system diseases and was hospitalized for pneumonia treatments since childhood. HRCT showed situs inversus totalis and imaging features of bronchiectasis (Fig. 2h). In addition, a CT scan of the paranasal sinus showed thickened mucosa of the bilateral maxillary sinus, ethmoidal sinus and sphenoid sinus (Fig. 2i). Patient 3 died at the age of 42 years old of lung failure. The clinical records of IV-3 were not accessible. According to his parents, IV-3 had recurrent lung infections since three years old. He was diagnosed with situs inversus and suffered from lung failure at the age of 38. After home oxygen therapy for six months, the patient died of lung failure. No PCD symptoms were observed in the consanguineous parents or Patient 3's daughter. No abnormal organ positions or bronchiectasis features were observed on CXR (Fig. 2b, c).

The detailed clinical manifestations and latest medical examination of each patient are summarized in Table 1.

**Table 1:** Phenotypic features of individuals with *DNAH5*

Characteristics	Patient 1	Patient 2	Patient 3
Known consanguinity	No	Yes	Yes
Gender	Female	Male	Male
Age (years)	0.6	39.0	41.0
High (cm)	62.7	162.0	164.0
Weight (kg)	7.3	61.5	53.5
Birth status	Full-term natural labor	Full-term natural labor	Full-term natural labor
Situs inversus	Yes	Yes	Yes
Neonatal respiratory distress	Yes	No	Yes
Respiratory symptom	Year-round wet cough	Intermittent wet cough	Year-round wet cough
Number of acute pneumonias	1	More than 20	NA
Respiratory microbiology	No	*Pseudomonas aeruginosa*	NA
Otitis media	Yes	No	Unknown
Hearing problems	No	No	Unknown
Sinusitis	Yes	Yes	Yes
Smell problems	Unknown	No	No
Nasal nitric oxide (nL/min)	8.1	9.9	NA
FVC	NA	62.7%	38.1%
FEV1, % pred	NA	62.2%	33.2%
HRCT	Normal	Bronchiectasis; Rhinosinusitis	Bronchiectasis; Rhinosinusitis
Lung failure	NA	NA	At 42 years old
Infertility	NA	Yes	No

*FEV1* forced expiratory volume in 1 s, *FVC* forced vital capacity, *NA* not available

### Ciliary Structural and Functional Analysis

Ultrastructural analysis of Patient 1 showed a shortened ODA. HSVA of her nasal cilia exhibited minimal residual, disorganized beating (Video S1). Ultrastructural analysis of Patient 2 and Patient 3 showed complete absence of ODA. Most frames in the videos showed completely immotile cilia (Video S2). A powerful beating stroke followed by a recovery stroke was observed in the healthy control (Video S3).

### Genetic Analysis and Validation

**Fig. 3:**
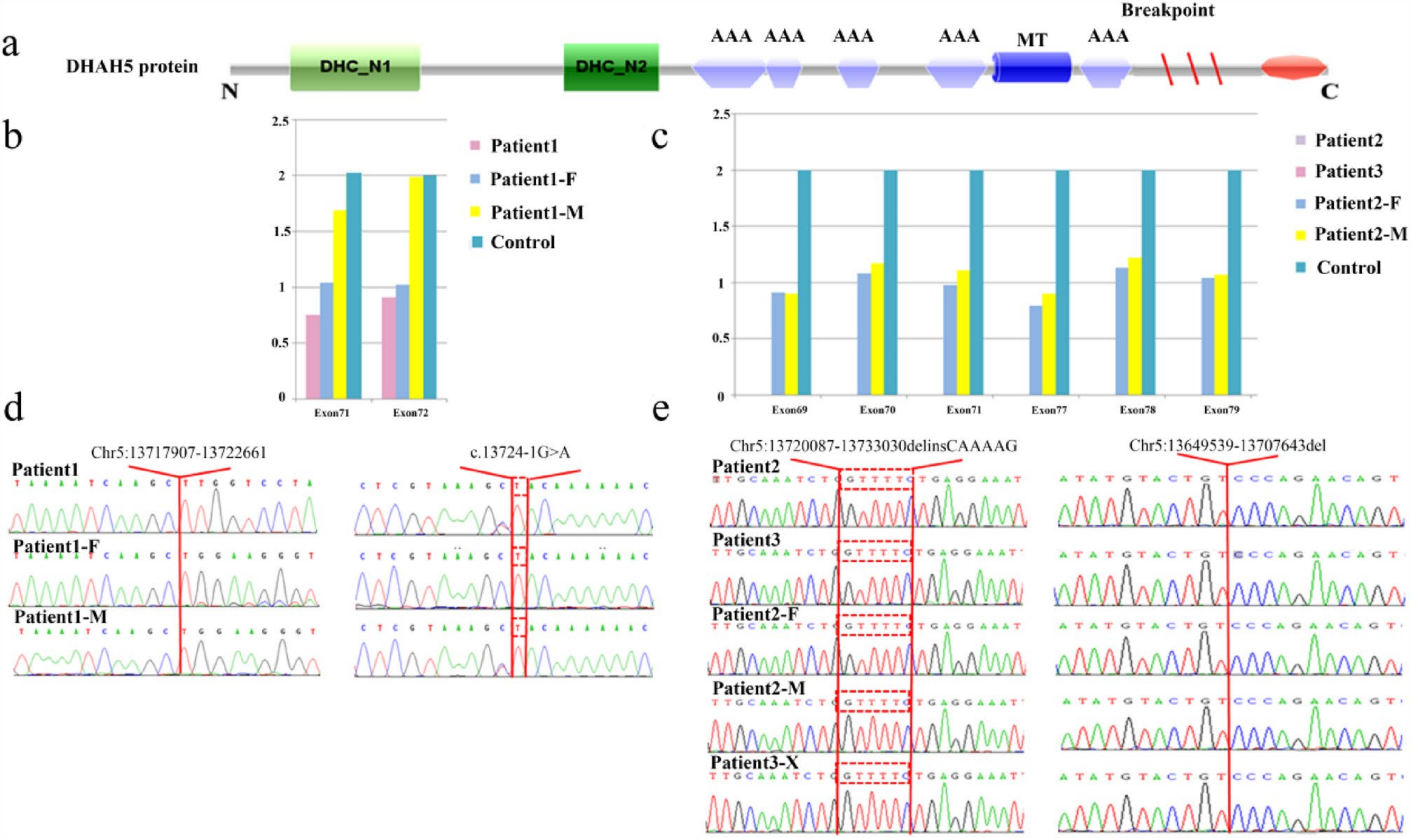
CNV analysis by qPCR and Sanger sequencing. **a** Schematic of the DNAH5 protein structure, with the red slash indicating the position of the CNVs. qPCR analysis of relative DNA content in whole blood from the control subject, Patient 1 (**b**), Patient 2 (**c**) and their family members. Sanger sequencing confirmed the CNVs at chr5:13717907-13722661 in Patient 1 (**d**) and chr5:13720087_13733030 and chr5:13649539_13707643 in Patient 2 (**e**)

The WES results were analyzed with an algorithm to detect CNVs. The positions of the CNVs identified in our study are shown in Fig. 3a. A CNV causing heterozygous deletions corresponding to exons 71–72 of *DNAH5* was suspected in Patient 1 by analysis. To verify the large deletions, qPCR was performed with DNA from the proband and her family members. Single copy loss was detected in Patient 1 and her father by qPCR (Fig. 3b). As WES only covers exonic regions, WGS was performed to specify the precise breakpoints. Eventually, chr5:13717907-13722661del was detected and confirmed by Sanger sequencing (Fig. 3d). Two CNVs causing homozygous deletions spanning exons 69–71 and exons 77–79 were suspected in Patient 2 and his eldest brother Patient 3 (Table 2); qPCR verified that exons 69–71 and exons 77–79 were homozygous deletions in Patient 2 and Patient 3 and heterozygous deletions in their consanguineous parents (Fig. 3c). This indicated an autosomal recessive inheritance pattern conforming to the genetic disposition of PCD. To specify the precise breakpoints of the two CNVs, WGS was performed. Ultimately, chr5:13720087_13733030delinsGTTTTC and chr5:13649539_13707643del were detected and confirmed by Sanger sequencing (Fig. 3e).

**Table 2:** *DNAH5* mutations in primary ciliary dyskinesia

Patient	Location	cDNA position	Zygosity	Allele	Type of variants	GnomAD, All	ACMG guideline	
							Class	Evidence
Patient 1	*Intron79*	c.13724-1G > A	Compound	Ma	Splice-site	None	LP	PVS1_Moderate; PM2_Supporting; PP4_Strong
	*Exon71-72*	4.7 kB del	Het	Pa	CNV	None	LP	PVS1_Moderate; PM2_Supporting; PP4_Strong
Patient 2	*Exon69-71*	58.1 kB	Hom	Pa, Ma	CNV	None	LP	PVS1_Moderate; PM2_Supporting; PP1; PP4_Strong
	*Exon77-79*	12.9 kB del	Hom	Pa, Ma	CNV	None	LP	PVS1_Moderate; PM2_Supporting; PP1; PP4_Strong
Patient 3	*Exon69-71*	58.1 kB	Hom	Pa, Ma	CNV	None	LP	PVS1_Moderate; PM2_Supporting; PP1; PP4_Strong
	*Exon77-79*	12.9 kB del	Hom	Pa, Ma	CNV	None	LP	PVS1_Moderate; PM2_Supporting; PP1; PP4_Strong

*ACMG* American College of Medical Genetics and Genomics, *CNV* Copy number variation, *LP* Likely pathogenic

**Fig. 4:**
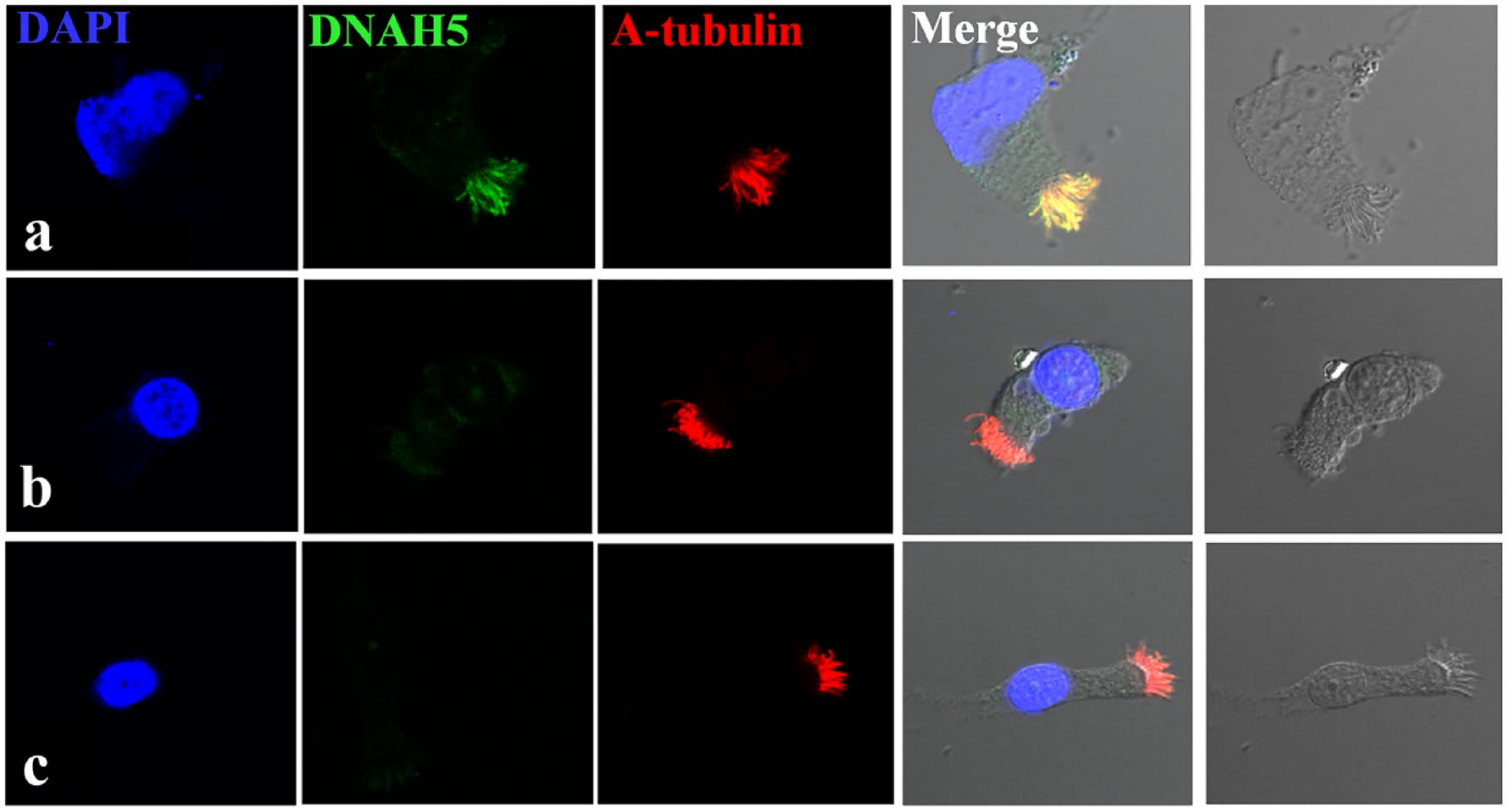
Subcellular localization of DNAH5 in respiratory epithelial cells. Axoneme-specific antibodies against acetylated α-tubulin (red) were used as the control. In respiratory epithelial cells from healthy probands (**a**), DNAH5 (green) localized predominantly along the entire length of the axonemes, as well as to the apical cytoplasm. In respiratory epithelial cells from individuals Patient 1 (**b**) and Patient 2 (**c**), DNAH5 was absent from the axoneme and markedly reduced in the apical cytoplasm

To further confirm the pathogenicity of the CNVs, we analyzed the cDNA of Patient 2. The consequence of chr5:13720087_13733030del was verified at the messenger ribonucleic acid (mRNA) level. It resulted in a reading frameshift after exon 68, followed by a premature stop codon at exon 72 (Fig. S1a). Subsequently, the ciliary protein of Patient 2 was extracted for western blotting. The level of DNAH5 protein expression was obviously decreased (Fig. S1b). Since Patient 1 is an infant, we regrettably obtained very few cilia samples. Therefore, cDNA sequencing and western blotting could not be performed on Patient 1. However, chr5:13717907-13722661del, which spans exons 71 and 72 of *DNAH5,* was believed to cause a 466-bp deletion at the mRNA level. The 466-bp deletion was predicted to cause a reading frameshift during translation, which could damage protein coding. In addition, c.13724-1G > A was predicted to cause a splicing aberration by scsnv. Finally, we studied DNAH5 localization in respiratory cells by IF and found a complete absence of DNAH5 in the ciliary axonemes of Patient 1 and Patient 2 compared with the healthy control (Fig. 4).

## Discussion

Our study recruited patients with situs inversus totalis, chronic sinusitis, and productive cough. HSVA, nNO, and TEM results suggested that these patients had high suspicion of PCD. A total of 115 patients have undergone genetic testing in our center. Through NGS and targeted CNV analysis, clinically significant single-nucleotide variants (SNVs) have been identified in 97 patients. CNVs have been detected in three patients. Subsequent qPCR confirmed the copy number abnormalities. WGS and Sanger sequencing were performed to specify and confirm the precise breakpoints. IF was performed to further support the pathogenicity. In addition, there are 15 cases where the carrier status is unknown in our cohort.

Several studies have systematically investigated associations between genotype and phenotype in PCD (Emiralioğlu et al. 2020; Pifferi et al. 2021; Shoemark et al. 2021; Blanchon et al. 2020). Individuals with *DNAH5* mutations have been reported to be phenotypically diverse (Shoemark et al. 2021; Blanchon et al. 2020). Consistent with a previous report, phenotypic diversity was observed in our cohort, even among siblings. Despite frequent respiratory infections, Patient 2 still had an apparently normal life at 39 years old with only mild to moderate impairment of pulmonary function. In contrast, his older brothers who carried identical mutations and were in the same living environment were not so fortunate; they all had more severe bronchiectasis and died from progressive lung failure at middle age. Data on the mortality of PCD are extremely limited. To the best of our knowledge, the two respiratory deaths in our study were the first reported in *DNAH5*-associated PCD.

In recent years, sequencing techniques and bioinformatics analysis have rapidly advanced. Currently, WES plays a very important role in PCD diagnosis in China. To date, over 50 genes have been reported to cause PCD (Wallmeier et al. 2020). However, the genetic basis of PCD remains unknown in approximately 30% of suspected patients. Routine WES analysis most often focuses on SNVs and short insertions and deletions. CNVs regrettably cannot be detected by routine WES analysis (Harel et al. 2018). Defined as genomic intervals that deviate from the normal diploid state, CNVs have been collectively detected in an estimated 12–16% of the human genome (Pös et al. 2021). However, the prevalence and clinical significance of CNVs in PCD-associated genes are unclear. Few studies have reported the relationship between CNVs and PCD in European, American and Japanese populations (Takeuchi et al. 2020; Marshall et al. 2015; Keicho et al. 2020). Marshall and colleagues reported that WES followed by targeted CNV analysis identified four of 45 (8.8%) PCD patients who harbored clinically significant CNVs (Marshall et al. 2015). Fassad reported that CNVs accounted for 4% of variants overall in PCD patients (Fassad et al. 2020).

In the present study, CNVs were predicted by analysis with WES data plus panelcn.MOPS, a reliable algorithm to detect CNVs with high sensitivity and specificity, and confirmed by qPCR and Sanger sequencing. Through this process, we identified three clinically significant CNVs in two Chinese families with PCD. Our study suggests that the complementary roles of WES and CNV analysis in the molecular diagnosis of PCD are clinically beneficial. Generally, the gold standard tool for CNV detection in diagnostic settings is microarray. Microarray is economical and fast for large CNVs, but it is not normally sensitive for small CNV events involving one or a few exons. On the other hand, microarrays can only detect the target region covered by the probes. The accuracy of WES in different regions varies according to sequencing depth. Therefore, CNVs detected based on WES data need to be further verified. However, WES allows for the identification of large and small CNVs in entire coding regions at once. It also permits the detection of both and CNVs concurrently, thus eliminating the need to use a range of different technologies in one patient and optimizing the diagnostic process (Royer-Bertrand et al. 2021).

## Conclusion

Our study provides a detailed description of Chinese PCD patients with CNVs of *DNAH5* and confirmed that CNVs play a valuable role in PCD caused by *DNAH5*.

### Supplementary Information

Below is the link to the electronic supplementary material.

Supplementary file1 (DOCX 15 KB)

Supplementary file2 (DOCX 15 KB)

Supplementary file3 (MP4 1945 KB)

Supplementary file4 (MP4 2502 KB)

Supplementary file5 (MP4 509 KB)

Supplementary file6 (DOCX 2923 KB)

## Data Availability

All data and material are available from the corresponding author on reasonable request.

## Abbreviations

ACMG: American College of Medical Genetics and Genomics
cn.MOPS: Copy number estimation by a mixture of Poissons
CNV: Copy number variation
DNA: Deoxyribonucleic acid
DNAH5: Dynein axonemal heavy chain 5
FEV1: Forced expiratory volume in the first second
HRCT: High-resolution computed tomography
HSVA: High-speed video analysis
IF: Immunofluorescence
mRNA: Messenger ribonucleic acid
NGS: Next-generation sequencing
nNO: Nasal nitric oxide
ODA: Outer dynein arms
PCD: Primary ciliary dyskinesia
qPCR: Quantitative real-time polymerase chain reaction
SNVs: Single-nucleotide variants
TEM: Transmission electron microscopy
WES: Whole-exome sequencing
WGS: Whole-genome sequencing

## References

[CR1] Aradhya S, Lewis R, Bonaga T, Nwokekeh N, Stafford A, Boggs B, Hruska K, Smaoui N, Compton JG, Richard G, Suchy S (2012). Exon-level array CGH in a large clinical cohort demonstrates increased sensitivity of diagnostic testing for Mendelian disorders. Genet Med.

[CR2] Blanchon S, Legendre M, Bottier M, Tamalet A, Montantin G, Collot N, Faucon C, Dastot F, Copin B, Clement A, Filoche M, Coste A, Amselem S, Escudier E, Papon JF, Louis B (2020). Deep phenotyping, including quantitative ciliary beating parameters, and extensive genotyping in primary ciliary dyskinesia. J Med Genet.

[CR3] Emiralioğlu N, Taşkıran EZ, Koşukcu C, Bilgiç E, Atilla P, Kaya B, Günaydın Ö, Yüzbaşıoğlu A, Tuğcu GD, Ademhan D, Eryılmaz Polat S, Gharibzadeh Hızal M, Yalçın E, Doğru D, Kiper N, Alikaşifoğlu M, Özçelik U (2020). Genotype and phenotype evaluation of patients with primary ciliary dyskinesia: first results from Turkey. Pediatr Pulmonol.

[CR4] Fassad MR, Patel MP, Shoemark A, Cullup T, Hayward J, Dixon M, Rogers AV, Ollosson S, Jackson C, Goggin P, Hirst RA, Rutman A, Thompson J, Jenkins L, Aurora P, Moya E, Chetcuti P, O'Callaghan C, Morris-Rosendahl DJ, Watson CM, Wilson R, Carr S, Walker W, Pitno A, Lopes S, Morsy H, Shoman W, Pereira L, Constant C, Loebinger MR, Chung EMK, Kenia P, Rumman N, Fasseeh N, Lucas JS, Hogg C, Mitchison HM (2020). Clinical utility of NGS diagnosis and disease stratification in a multiethnic primary ciliary dyskinesia cohort. J Med Genet.

[CR5] Guan Y, Yang H, Yao X, Xu H, Liu H, Tang X, Hao C, Zhang X, Zhao S, Ge W, Ni X (2021). Clinical and genetic spectrum of children with primary ciliary dyskinesia in China. Chest.

[CR6] Guo Z, Chen W, Wang L, Qian L (2020). Clinical and genetic spectrum of children with primary ciliary dyskinesia in China. J Pediatr.

[CR7] Harel T, Lupski JR (2018). Genomic disorders 20 years on-mechanisms for clinical manifestations. Clin Genet.

[CR8] Hornef N, Olbrich H, Horvath J, Zariwala MA, Fliegauf M, Loges NT, Wildhaber J, Noone PG, Kennedy M, Antonarakis SE, Blouin JL, Bartoloni L, Nüsslein T, Ahrens P, Griese M, Kuhl H, Sudbrak R, Knowles MR, Reinhardt R, Omran H (2006). DNAH5 mutations are a common cause of primary ciliary dyskinesia with outer dynein arm defects. Am J Respir Crit Care Med.

[CR9] Keicho N, Hijikata M, Morimoto K, Homma S, Taguchi Y, Azuma A, Kudoh S (2020). Primary ciliary dyskinesia caused by a large homozygous deletion including exons 1–4 of DRC1 in Japanese patients with recurrent sinopulmonary infection. Mol Genet Genom Med.

[CR10] Klambauer G, Schwarzbauer K, Mayr A, Clevert DA, Mitterecker A, Bodenhofer U, Hochreiter S (2012). cn.MOPS: mixture of Poissons for discovering copy number variations in next-generation sequencing data with a low false discovery rate. Nucleic Acids Res.

[CR11] Li H, Durbin R (2009). Fast and accurate short read alignment with Burrows–Wheeler transform. Bioinformatics.

[CR12] Li H, Handsaker B, Wysoker A, Fennell T, Ruan J, Homer N, Marth G, Abecasis G, Durbin R (2009). 1000 Genome project data processing subgroup. The sequence alignment/map format and SAMtools. Bioinformatics.

[CR13] Lucas JS, Barbato A, Collins SA, Goutaki M, Behan L, Caudri D, Dell S, Eber E, Escudier E, Hirst RA, Hogg C, Jorissen M, Latzin P, Legendre M, Leigh MW, Midulla F, Nielsen KG, Omran H, Papon JF, Pohunek P, Redfern B, Rigau D, Rindlisbacher B, Santamaria F, Shoemark A, Snijders D, Tonia T, Titieni A, Walker WT, Werner C, Bush A, Kuehni CE (2017). European Respiratory Society guidelines for the diagnosis of primary ciliary dyskinesia. Eur Respir J.

[CR14] Marshall CR, Scherer SW, Zariwala MA, Lau L, Paton TA, Stockley T, Jobling RK, Ray PN, Knowles MR, Hall DA, Dell SD, Kim RH, FORGE Canada Consortium (2015). Whole-exome sequencing and targeted copy number analysis in primary ciliary dyskinesia. G3 (bethesda).

[CR15] McKenna A, Hanna M, Banks E, Sivachenko A, Cibulskis K, Kernytsky A, Garimella K, Altshuler D, Gabriel S, Daly M, DePristo MA (2010). The Genome Analysis Toolkit: a MapReduce framework for analyzing next-generation DNA sequencing data. Genome Res.

[CR16] Olbrich H, Häffner K, Kispert A, Völkel A, Volz A, Sasmaz G, Reinhardt R, Hennig S, Lehrach H, Konietzko N, Zariwala M, Noone PG, Knowles M, Mitchison HM, Meeks M, Chung EM, Hildebrandt F, Sudbrak R, Omran H (2002). Mutations in DNAH5 cause primary ciliary dyskinesia and randomization of left-right asymmetry. Nat Genet.

[CR17] Pifferi M, Bush A, Mulé G, Gracci S, Fonnesu R, Michelucci A, Cangiotti A, Caligo MA, Miccoli M, Boner AL, Peroni D (2021). Longitudinal lung volume changes by ultrastructure and genotype in primary ciliary dyskinesia. Ann Am Thorac Soc.

[CR18] Pös O, Radvanszky J, Buglyó G, Pös Z, Rusnakova D, Nagy B, Szemes T (2021). DNA copy number variation: main characteristics, evolutionary significance, and pathological aspects. Biomed J.

[CR19] Povysil G, Tzika A, Vogt J, Haunschmid V, Messiaen L, Zschocke J, Klambauer G, Hochreiter S, Wimmer K (2017). panelcn.MOPS: copy-number detection in targeted panel data for clinical diagnostics. Hum Mutat.

[CR20] Raidt J, Wallmeier J, Hjeij R, Onnebrink JG, Pennekamp P, Loges NT, Olbrich H, Häffner K, Dougherty GW, Omran H, Werner C (2014). Ciliary beat pattern and frequency in genetic variants of primary ciliary dyskinesia. Eur Respir J.

[CR21] Richards S, Aziz N, Bale S, Bick D, Das S, Gastier-Foster J, Grody WW, Hegde M, Lyon E, Spector E, Voelkerding K, Rehm HL (2015). ACMG Laboratory Quality Assurance Committee. Standards and guidelines for the interpretation of sequence variants: a joint consensus recommendation of the American College of Medical Genetics and Genomics and the Association for Molecular Pathology. Genet Med.

[CR22] Royer-Bertrand B, Cisarova K, Niel-Butschi F, Mittaz-Crettol L, Fodstad H, Superti-Furga A (2021). CNV detection from exome sequencing data in routine diagnostics of rare genetic disorders: opportunities and limitations. Genes (basel).

[CR23] Shoemark A, Rubbo B, Legendre M, Fassad MR, Haarman EG, Best S, Bon ICM, Brandsma J, Burgel PR, Carlsson G, Carr SB, Carroll M, Edwards M, Escudier E, Honoré I, Hunt D, Jouvion G, Loebinger MR, Maitre B, Morris-Rosendahl D, Papon JF, Parsons CM, Patel MP, Thomas NS, Thouvenin G, Walker WT, Wilson R, Hogg C, Mitchison HM, Lucas JS (2021). Topological data analysis reveals genotype–phenotype relationships in primary ciliary dyskinesia. Eur Respir J.

[CR24] Takeuchi K, Xu Y, Kitano M, Chiyonobu K, Abo M, Ikegami K, Ogawa S, Ikejiri M, Kondo M, Gotoh S, Nagao M, Fujisawa T, Nakatani K (2020). Copy number variation in DRC1 is the major cause of primary ciliary dyskinesia in the Japanese population. Mol Genet Genom Med.

[CR25] Wallmeier J, Nielsen KG, Kuehni CE, Lucas JS, Leigh MW, Zariwala MA, Omran H (2020). Motile ciliopathies. Nat Rev Dis Primers.

[CR26] Wang L, Zhao X, Liang H, Zhang L, Li C, Li D, Meng X, Meng F, Gao M (2021). Novel compound heterozygous mutations of DNAH5 identified in a pediatric patient with Kartagener syndrome: case report and literature review. BMC Pulm Med.

[CR01] Zariwala MA, Knowles MR, Leigh MW (1993–2023) Primary Ciliary Dyskinesia. 2007 Jan 24 [Updated 2019 Dec 5]. In: Adam MP, Mirzaa GM, Pagon RA, et al (eds) GeneReviews® [Internet]. University of Washington, Seattle, Seattle (WA). Available from: https://www.ncbi.nlm.nih.gov/books/NBK1122/20301301

