# Supplementary material for: Next-Generation Sequencing-Based Copy Number Variation Analysis in Chinese Patients with Primary Ciliary Dyskinesia Revealed Novel DNAH5 Copy Number Variations
Source: Phenomics. 2024 Feb 22;4(1):24–33. doi: 10.1007/s43657-023-00130-0 (PMC11003934; doi:10.1007/s43657-023-00130-0)
Supplement: Supplementary file 1 — Supplementary file1 (DOCX 15 KB) [file 43657_2023_130_MOESM1_ESM.docx]

**Table S1** The methods used in this study

| Methods | Patient 1 | Patient 1’s parents | Patient 2 | Patient 3 | Patient 2’s parents | Patient 3’s daughter |
| --- | --- | --- | --- | --- | --- | --- |
| Know Phenotypic assessment | Yes | Yes | Yes | Yes | Yes | Yes |
| Lung function | NA | NA | Yes | Yes | NA | NA |
| HRCT | Yes | NA | Yes | Yes | NA | NA |
| Nasal nitric oxide | Yes | NA | Yes | NA | NA | NA |
| TEM | Yes | NA | Yes | Yes | NA | NA |
| HSVA | Yes | NA | Yes | Yes | NA | NA |
| WES | Yes | Yes | Yes | Yes | Yes | NA |
| QPCR | Yes | Yes | Yes | Yes | Yes | NA |
| WGS | Yes | NA | Yes | NA | NA | NA |
| Sanger sequencing | Yes | Yes | Yes | Yes | Yes | Yes |
| cDNA analysis | NA | NA | Yes | NA | NA | NA |
| WB | NA | NA | Yes | NA | NA | NA |
| IF | Yes | NA | Yes | NA | NA | NA |

HRCT, forced expiratory volume in 1 second; HSVA, not available; IF, immunofluorescence; QPCR, quantitative real-time polymerase chain reaction; TEM, forced vital capacity; WB, western blot; WES, whole exome sequencing; WGS, whole genome sequencing;
