# Supplementary material for: Next-Generation Sequencing-Based Copy Number Variation Analysis in Chinese Patients with Primary Ciliary Dyskinesia Revealed Novel DNAH5 Copy Number Variations
Source: Phenomics. 2024 Feb 22;4(1):24–33. doi: 10.1007/s43657-023-00130-0 (PMC11003934; doi:10.1007/s43657-023-00130-0)
Supplement: Supplementary file 2 — Supplementary file2 (DOCX 15 KB) [file 43657_2023_130_MOESM2_ESM.docx]

**Table S2** Summary of quantitative real-time polymerase chain reaction primers

| Primer Name | Sequence | primer length | product length（genomic） |
| --- | --- | --- | --- |
| Patient1-DNAH5-e71-F | GTTTGCCTGACAGGGTTACTGT | 22 | 179 |
| Patient1-DNAH5-e71-R | AACACCTTCGGCATATTTTTCTCC | 24 |  |
| Patient1-DNAH5-e72-F | TTGTACATGATGCGTTCCGC | 20 | 176 |
| Patient1-DNAH5-e72-F | GGAAACTCCTGTAGACTCGCAA | 22 |  |
| Patient2,Patient3-DNAH5-e69-F | AGGAGGTGCCTCATTAGACCTTA | 23 | 100 |
| Patient2,Patient3-DNAH5-e69-R | TCTGAGTTTGCTAAGTTCCACCA | 23 |  |
| Patient2,Patient3-DNAH5-e70-F | AGGAACCTCTTCCAAATGCCTAT | 23 | 109 |
| Patient2,Patient3-DNAH5-e70-R | TATGGACTTTTTACCTGGGCGA | 22 |  |
| Patient2,Patient3-DNAH5-e71-F | GGAGGAATCTGATCCACGGAC | 21 | 185 |
| Patient2,Patient3-DNAH5-e71-R | TATTCAGCCTTACGTTCGCCA | 21 |  |
| Patient2,Patient3-DNAH5-e77-F | TCTTGGATTTCTAGTACACTGGGTT | 25 | 101 |
| Patient2,Patient3-DNAH5-e77-R | CAAAAGCAGTGAGGTCGGC | 19 |  |
| Patient2,Patient3-DNAH5-e78-F | CCAACAAAGGCTGGGCTCT | 19 | 143 |
| Patient2,Patient3-DNAH5-e78-R | GTTCCTCTTGTCCCAGCCAG | 20 |  |
| Patient2,Patient3-DNAH5-e79-F | AACTACATTGCCGCTGTGGAT | 21 | 106 |
| Patient2,Patient3-DNAH5-e79-R | GGACACTCCCCACATGTTACTT | 22 |  |
