# Supplementary material for: Next-Generation Sequencing-Based Copy Number Variation Analysis in Chinese Patients with Primary Ciliary Dyskinesia Revealed Novel DNAH5 Copy Number Variations
Source: Phenomics. 2024 Feb 22;4(1):24–33. doi: 10.1007/s43657-023-00130-0 (PMC11003934; doi:10.1007/s43657-023-00130-0)
Supplement: Supplementary file 6 — Supplementary file6 (DOCX 2923 KB) [file 43657_2023_130_MOESM6_ESM.docx]

**
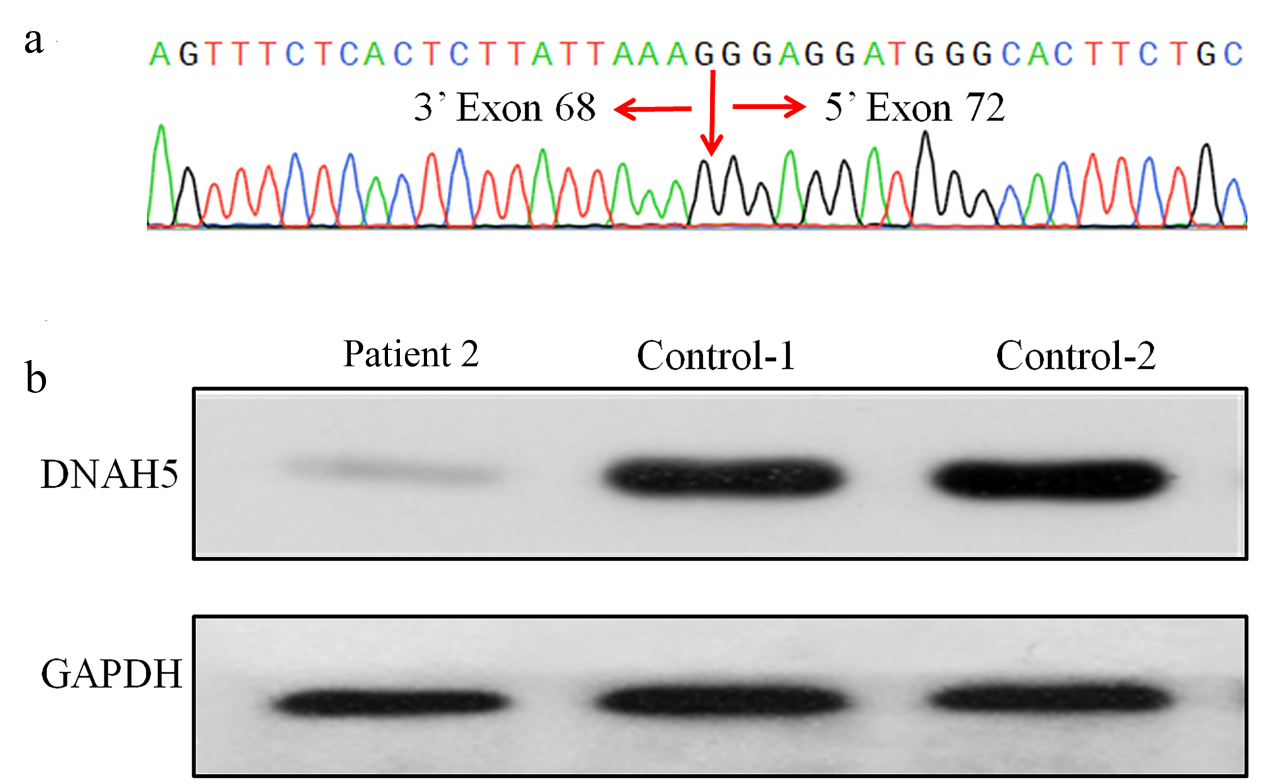
**

**Fig. S1**

(a) Sanger sequencing of cDNA from Patient 2’s nasal sample. (b) Western blot analysis of protein extracts from Patient 2 nasal samples showed significantly reduced expression of DNAH5
